# Digital Inequalities in Cancer Care Delivery in India: An Overview of the Current Landscape and Recommendations for Large-Scale Adoption

**DOI:** 10.3389/fdgth.2022.916342

**Published:** 2022-06-27

**Authors:** Ramachandran Venkataramanan, Akash Pradhan, Abhishek Kumar, Arnie Purushotham, Mohannad Alajlani, Theodoros N. Arvanitis

**Affiliations:** ^1^Institute of Digital Healthcare, WMG, University of Warwick, Coventry, United Kingdom; ^2^Research Division, Karkinos Healthcare, Mumbai, India; ^3^School of Cancer and Pharmaceutical Sciences, King's College London, London, United Kingdom

**Keywords:** cancer care, digital divide, technology, pandemic, India, policy

## Abstract

**Introduction:**

COVID-19 pandemic has caused major disruptions to delivery of various cancer care services as efforts were put to control the outbreak of the pandemic. Although the pandemic has highlighted the inadequacies of the system but has also led to emergence of a new cancer care delivery model which relies heavily on digital mediums. Digital health is not only restricted to virtual dissemination of information and consultation but has provided additional benefits ranging from support to cancer screening, early and more accurate diagnosis to increasing access to specialized care. This paper evaluates the challenges in the adoption of digital technologies to deliver cancer care services and provides recommendation for large-scale adoption in the Indian healthcare context.

**Methods:**

We performed a search of PubMed and Google Scholar for numerous terms related to adoption of digital health technologies for cancer care during pandemic. We also analyze various socio-ecological challenges—from individual to community, provider and systematic level—for digital adoption of cancer care service which have existed prior to pandemic and lead to digital inequalities.

**Results:**

Despite encouraging benefits accruing from the adoption of digital health key challenges remain for large scale adoption. With respect to user the socio-economic characteristics such as age, literacy and socio-cultural norms are the major barriers. The key challenges faced by providers include regulatory issues, data security and the inconvenience associated with transition to a new system.

**Policy Summary:**

For equitable digital healthcare, the need is to have a participatory approach of all stakeholders and urgently addressing the digital divide adequately. Sharing of health data of public and private hospitals, within the framework of the Indian regulations and Data Protection Act, is critical to the development of digital health in India and it can go a long way in better forecasting and managing cancer burden.

## Introduction

India has launched several policies and programs to address the problem of cancer care in the country. Most recently, the government of India has introduced mandatory screening for three types of cancer-breast, cervical and oral- at the Primary Health Center (PHC) across 100 districts through the National Programme for the Prevention and Control of Cancer, Diabetes, Cardiovascular Diseases and Stroke (NPCDCS) to detect cancer at early stages (1). Unfortunately, COVID-19 pandemic has caused major disruptions to delivery of various cancer care services as efforts were put to control the outbreak of the pandemic (2). Recently, a cohort study based on data from 41 cancer centers reveal that a majority of patients suffering from cancer have been unable to register, follow up for diagnostic check-ups and undergo chemotherapy due to the pandemic (3). Although the pandemic has highlighted the inadequacies of the system but has also led to emergence of a new cancer care delivery model which relies heavily on digital mediums.

In this context, the term digital health is defined by World Health Organization (WHO) as “a broad umbrella term encompassing eHealth (which includes mHealth), as well as emerging areas, such as the use of advanced computing sciences in “big data”, genomics and artificial intelligence” (4). While e-health is defined as the use of information and communication technology such as computer, internet and mobile phones to facilitate delivery of health care services (4). Mobile health (mhealth) is a subset of e-health and encompasses the use of mobile devices to overcome the challenges related to delivery of health care services (4). The definition of digital health has evolved over time and now include a wide range of digital technologies which are used to provide distant consultation and to store records electronically such as telemedicine, digital health records, clinical decision support system (CDSS) and web-based health services (5). Digital health services have been instrumental in improving the support for successful cancer screening, manage symptoms post-treatment, and provide support during treatment and communication between patients and providers (6–8).

Although global health has improved significantly due to digitization: from early and more accurate diagnosis of cancer to increasing access to specialized care, capturing essential information at the point of care for better public health interventions, information delivering personalized care through mHealth, and managing healthcare services in community settings (9). However, such advancements cannot be generalized for low public health-resourced countries like India. Notably, India has seen a substantial increase in cancer cases over the last few decades. However, a considerable part of the Indian population, particularly the low-income segment, remains under-diagnosed and under-treated given limited accessibility and affordability for cancer care services at both public and private facilities. In particular, households do not generally seek health care from public sector because of poor quality of care, distance to the facility and long waiting time at government facilities (10). Lack of specialized services drives patients to seek services at private facilities which are the dominant players in the Indian health care sector. However, lack of regulation and non-uniformity in cost of delivery of services at private facilities translate into lack of accountability and high out of pocket expenditure (11). Furthermore, only around 5–15% of patients are diagnosed in the early stages of the disease and more than 70% of cancer cases present in locally advanced stages leading to poor survival (12). The mortality to incidence ratio of 0.68 in India is far higher than that in very high human development index (HDI) countries (0.38) and high HDI countries (0.57) (13). The application of advanced technological innovations, in low and middle-income countries (LMICs), is relatively low due to limited resources and opportunities to leverage the benefits of digital health (14, 15).

Owning to the pandemic, the Government of India released guidelines to enable Registered Medical Practitioners to Provide Healthcare using Telemedicine (16). Although, the use of information technology in healthcare in India has been envisaged during the 1990 but service providers have been reluctant to adopt the technology. With the risk of getting infected and restricted mobility during the pandemic both the providers and patients were compelled to use the digital mediums for consultation and follow ups. In this regard, the use of social media platforms (for example WhatsApp and Facebook) to monitor the condition of cancer patient and to provide psychological support proved effective (17). Apart from virtual dissemination of information, tele-consultation and social support through internet, the information technology can be used for cancer patient navigation by increasing access and reducing delays (18). In addition, Electronic Health Record (EHR) which is defined as “a longitudinal health record with entries by healthcare practitioners in multiple sites where care is provided” (19) have proved an effective solution to improve quality care and reduce medical errors. Digital formatting associated with EHR can be used to share information over secure networks, seamless tracking of health status and outcomes of the patient, ability to trigger warnings and reminders, send and receive results and reports; and ability to generate bills more accurately in a shorter span of time.

Clearly, investing in digital health in resource-constrained countries can reduce direct care costs, enhance the access to health services, and improve health outcomes (20). A systematic review of digital interventions for Non-Communicable Diseases (NCDs) in India, by Hossain et al., reported an insufficient number of digital interventions compared to the burden of NCDs in India. Unlike in many developed nations, digital health interventions for NCDs have not reached their fullest potential in India (21). Apart from resource constraints, the federated structure of India's health system poses challenges for adopting uniform guidelines with respect to information technology for cancer care as health is a State subject. The fragmentation between public and private sectors with respect to technology and lack of coo-ordination implies that integration of the system as a whole might not be feasible. Moreover, apart from socio-economic inequalities in usage of mobile phone and internet, the storage of data and interoperability remain key issues (22).

This study highlights the digital inequalities in cancer care in India related to provision as well as utilization of health care services which will provide insights for providing guidelines regarding patient-centered technologically interventions.

## Methods

A rapid review of literature on digital inequalities in cancer care over a 5 week period was undertaken in 2021. We conducted the rapid review to provide timely evidence on the challenges which might prevent successful implementation of the digital programs. Although the process to conduct rapid review is quite similar to systematic review however the review are less rigorous as certain steps might be omitted due to time constraint.

### Information Sources

We primarily searched PubMed. Two authors were involve in conducting the review. The search was not restricted to these two databases, we also searched the gray literature via internet search engine such as Google Scholar. We specifically looked for two types of documents. First published articles in peer reviewed journal which documented the challenges related to adoption of digital health. Second, we also looked for WHO guidelines on digital health as well as the government policy documents related to use of telemedicine in context of India.

### Search Terms

Search was conducted with an aim to identify the challenges faced by health seekers to adopt digital health for seeking cancer care services as well as health providers to adopt the new technology. The key search terms included a combination of terms such as “cancer care,” “technology,” “mhealth,” “India,” “digital,” “inequalities,” “pandemic,” and “gaps.”

### Synthesis

The challenges related to adoption and digitization of cancer related services were classified into four categories: individual level, community level, provider level and systematic level. The classification was carried by one reviewer on the basis of the previous peer reviewed articles as well as government policy reports related to digital interventions which were identified during the search process.

The initial analysis was undertaken by one author with an aim to understand the consistency with which certain challenges were cited across the shortlisted studies. Iterative discussion took place to finalize the categories. The individual level challenges were identified as age and gender divide, human behavior and literacy levels. The individual level challenges could arise because of affordability, inability to use technology and fear to make a transition to a new platform. Community level challenges are related to customs and norms as well as resource constraints and geographical location which might determine usage of the technology. Similarly, provider level challenges are related to lack of capacity, infeasibility and data management issues.

The key systematic challenges were regulatory issues, data quality issues and database and leakage. Although the challenges across different categories might seem similar however they differ significantly with respect to the magnitude. While individual and provider level challenges are at the micro level and pertain to the characteristics of health seekers and health provider, the community and systematic level challenges are at the macro level and reflect the functioning of the system.

## Results

Socio-ecological challenges starting from individual to systematic level exist for the digitization of cancer care service resource-constrained countries like India.

### Individual Level

#### Age and Gender Divide

The use of digital health is particularly relevant for young adults, since they are the pervasive users of technology. Findings indicate that the older adults are less likely than younger adults to use computers and the World Wide Web and that demographic factors, gender, computer anxiety, and cognitive abilities are important predictors of the use of these technologies (23). Some populations are marginalized, like people with low literacy or elderly adults who may not have similar digital behavior as young adults with higher literacy or behavior in adopting new technologies in a faster way (24). In addition, access to mobile phones is highly skewed with women less likely to own a mobile phone particularly in developing countries (25, 26). The gender gap in access to mobile phones has been found to be associated with lower utilization of health care services (27).

#### Human Behavior

The pattern of using digital technologies may depend on individual behavioral factors. Some studies highlight that, in a geography like India, the human factors, rather than lack of resources and technology, are often the main obstacle for the delivery of digital cancer care services, like tele-oncology (28). Changing the mindset of the people involved is essential for implementing better technology (29).

#### Literacy

The pervasiveness of mobile phone ownership has made messaging, through short message service (SMS), the most optimal format to communicate with many patients. Mobile phones are increasingly being used by the government to deliver social, economic and health services. Higher literacy levels have shown higher SMS literacy. Wide differentials are observed in ability of women to read SMS with SMS literacy being high in north-eastern parts of India (30).

Ability to read text messages is an important factor for use of health services. Mobile phone ownership among women and use of maternal services shows strong evidence on the effectiveness and cost-effectiveness of maternal mobile messaging initiatives in India (31).

However, literacy levels are not uniform in different states across India. In such cases, households, that have not managed to acquire a mobile phone, are increasingly pressured to do so, in order to maintain the same level of healthcare access. It is simply expected of everyone to own a mobile phone and use it for accessing services. In such situations, technology adoption stops being a free choice.

### Community-Level

#### Geographical Divide

Availability of digital technologies may differ across populations and geographies, affecting the implementation of advanced technological innovations (32). For example, digital applications requiring constant internet access may not achieve the desired level of success among rural populations, who predominantly use incompatible devices or have limited access to high-speed internet. Failure of continuous internet connection is a major bottleneck for real-time digital capture of cancer screening programs in rural and hard-to-reach settings in India.

In India, the growth of digital interventions for NCDs is mostly observed in the urban areas. Use of mobile phones for delivering NCD services is associated with ownership of mobile phones and geographic levels have an important bearing in the distribution of mobile phone ownership in India (25). As per the census 2011, 68.84% of India's total population live in rural areas, hence, urban-centered growth may not help to improve population health outcomes (33). In addition, rural settings lack adequate infrastructure, access to healthcare, skilled workforce, and quality services (34). Technological advancements, like digital health services, may be a greater requirement in the rural settings which can bridge the healthcare gaps.

#### Socio-Cultural Divide

The generic online resources may not be applicable to entire population. Interventions which work among a certain population may not be recommended without evaluating the appropriateness of those applications. The diversity amongst Indian population requires bespoke models of digital interventions.

Language could become the biggest barrier. The availability of smart phones and low data prices has advantages such as ease of search and access to information. However, the internet application at present are supported by only a few regional languages. In fact, English maintains a hegemony over both internet and digital applications. It is essential that knowledge is accessible and open to more users who speak a different language, especially information related to health.

#### Socio-Economic Divide

For many, the obstacles are mainly socio-economic in nature. In a technologically developing nation like India, economic structures enforce differential access to information. The cost of internet data and the devices worsen the digital divide. Some people cannot afford to own and subscribe to/recharge a phone service on a regular basis, due to various financial reasons.

During the COVID-19 pandemic, acceleration and increasing adoption of digital healthcare and tele-medicine has taken off, but with uneven and widening socioeconomic inequalities with rural areas suffering more than urban settings. The COVID-19 epidemic has emphasized taking digital technology into the rural areas to be able to provide better and evenly accessible healthcare in remote areas. Despite the fact that the digital health utilization in the last few months has substantially improved compared to the last several years, there is an increasing digital divide between urban and rural areas, with a risk of creating a new class of digitally poor citizens.

### Provider-Level

#### Lack of Adequate Capacity

A critical lack of skills and capacities among healthcare providers is a major concern for digital health. Neither is digital health widely taught in medical schools or health educational settings or in in-service reorientation programs, nor is there any professional training for individual practitioners and institutional delivery of digital health services. As a result, there is a translational gap at the provider level, which may affect leveraging the benefits of digitization in cancer care services (32).

#### Data Management

Digitizing health records and patient profiles can improve patient care and reduce time spent on back-office tasks. However, outside of a few urban pockets, India's medical professionals have not yet embraced electronic health records (EHRs) (35). EHR systems must be designed with an eye on adhering to government standards and being flexible enough to clean and analyze data for insights.

#### Lack of Adoption

In India, especially in the rural areas, there are many independent practitioners in allopathic and the Indian systems of medicines who run small dispensaries, and there is no practice of even storing patient data on computers. For most of them, it is not a feasible option to enter data in computers on their own or engage data entry operators, merely to comply with the digitization protocols (36).

### Systematic Level

#### Regulatory Issues

The Indian public health system is pluralistic, hence, many formal and informal providers may offer healthcare services that are poorly regulated and standardized. There is not enough emphasis on the policies or regulatory measures on the digital health development across public health systems. Developing and implementing digital tools, in such contexts, may become an isolate effort by individual providers or for-profit healthcare institutions, a situation that may result in further inequalities, unorganized development of technological innovations, and poor quality of digital interventions, while incurring a higher cost to the users, providers, and society.

#### Data Quality Issues

Poor quality of case finding and data collection on cancer related information is also a major issue. The vast size, diversity in geography and ethnicity, and lack of infrastructure in India presents an enormous challenge to evaluate the burden of cancer, which has consequences for policymakers. Inadequate and unrepresentative cancer data precludes policymakers from having a transparent understanding of the size and trajectory of the problems they face (37).

There is no dispute that capturing patient data and digitizing it could help patients, health professionals, healthcare facilities, and researchers.

#### Database and Leakage

The National Cancer Registry Program is collecting and publishing cancer registry data. This will help stakeholders to optimize screening strategies for population at high-risk for certain types of cancer in different parts of the country. In considering progress in dealing with cancers, it is usual for us to consider incidence, prevalence, mortality, and survival. The data pertaining to the latter two is sorely lacking from Indian cancer centers (38). It is important for each center to prospectively maintain databases for all patients so that key outcome measures are available to the policy makers.

Cybersecurity is critical in the healthcare delivery, as millions of digital health records are being produced every day. In the latest data leak related to users in India, over a million medical records and 121 million medical images of Indian patients have been leaked online to be freely accessible by anyone (39).

## Recommendations

### Recommendations for Digital Technologies Integration in Indian Healthcare Context for Delivering of Cancer Care Services

The needs of service providers in LMICs, like India, are unique. Technological acceptance and integration can be achieved, if the technological offerings are easy to use and the benefits are understood, cost-effective and secured. The use of digital channels can help in improving cancer care outcomes across the spectrum of care—from prevention, early detection, diagnosis, staging, treatment and palliative care. We offer the following recommendation to those planning at develop a digital initiative in cancer care in LMICs, like India.

### Tele-Oncology

The urban-rural health divide can result from poor access and communication between patients and providers. Patients from rural areas especially those who face transportation issues may benefit from tele-medicine. The adoption and pervasiveness of mobile technology have expanded the scope of tele-medicine. One notable example has been the emergence of tele-dermatology for skin cancer screening/triage. The combination of tele-dermoscopy with clinical tele-consultation resulted in an improved sensitivity and specificity of 93 and 96%, respectively, compared with an in-person dermatology consultation control group (40). One such study, conducted by Kroenke et al., to determine whether centralized telephone-based care management coupled with automated symptom monitoring improved depression and pain, showed improved pain and depression outcomes in cancer patients receiving care (41).

### Distributed Architecture

There are few emerging hub-and-spoke models for delivering care in cancer with an integrated digital infrastructure for a seamless continuum of care. The distributed cancer care model, developed by the Tata Trusts (India's oldest and largest non-sectarian philanthropic organization), is aiming to create a technology-enabled patient-centric cancer institutions across India, for delivering uniform, high quality, accessible and affordable care closer to patients' homes (42).

Datta and Rajasekar proposed the creation of an integrated three-tier radiotherapy service, which consists of primary, secondary, and tertiary radiotherapy centers—coordinated through a tele-radiotherapy network (43).

### Training of Allied Health Professionals

The Central Government, in the 2018 budget, announced the Ayushman Bharat program, whereby Health and Wellness Centers are envisioned to provide comprehensive primary healthcare, including services for NCDs. These services consist of management and control of oral, breast and cervical cancers, to the entire population. To make the scheme work, the government has created a digital platform as an essential technology backbone (44). The screening and management shall be done by the front-line health workers, who are empowered with technological solutions. It will capture data from screening to referral, diagnosis, treatment and follow-up activities and connect with health workers, doctors and decision-makers in a single, integrated platform (42).

### Focus on Local Factors

For large scale adoption of a new systems, it is essential for the end users to perceive the value addition, and see the gaps that it fills and problems it addresses. Hence, the engagement of the end users in developing a solution is critical. All the digital interventions must have a grass-root, “bottom up” approach. Keeping the end users' perspective in mind, while adopting digital systems, is essential for effective implementation.

### m-Health Solutions

The increasing mobile phone penetration in India, particularly smart phone penetration, provides unprecedented opportunities for m-health in India. Adopting digital solutions across the care pathway of prevention, diagnosis and cure can transform the delivery of cancer care services, yielding improved outcomes.

SMS text messages can aid in the delivery of a wide variety of information directly to patients. SMS is convenient for a patient in many ways. There is no dependence on internet connection. As per the 2015–16 National Family Health Survey, 61.8% of the urban and 36.9% of the rural women population in India have a mobile phone that they themselves use (10).

Several authors have reported that text messaging may possibly be the preferred method for communication about screening tests (45, 46). Text messaging has been shown to improve attendance in mammography screening programs both in remote locations and in higher-risk populations (47). Similarly, for cervical and colorectal cancers text messaging interventions have been shown to improve screening participation (48).

## Conclusion

Adopting the digital cancer care models during the outbreak of the pandemic have showed that digital tools could prove effective to provide care to patients who might not be able to visit the health facilities. Implementation of digital models will require installation of the software, converting the existing records to electronic format and training of the health workers.

Furthermore, all the digital interventions must have a grass-root, “bottom up” approach. Keeping the end users' perspective in mind, while adopting digital systems, is essential for effective implementation. Developments in digital infrastructure offer an unprecedented opportunity to overcome the conventional barriers of geography, infrastructure, and culture. Digital health has transformed the traditional healthcare system, where empowered patients can take informed decisions, customized to their needs. However, the research about guidelines to put in place for its use, standardization of process and the steps to protect privacy of the patients are some of the emerging issues which will take considerable time of the various stakeholders in future.

## Author Contributions

RV, APr, and TA contributed to conception and design of the study. APr organized the database. RV wrote the first draft of the manuscript. APr, AK, APu, MA, and TA wrote sections of the manuscript. AK, APu, MA, and TA provided revisions to the manuscript. APr and AK provided stylistic/grammatical revision to the manuscript. All authors contributed to manuscript revision, read, and approved the submitted version.

## Conflict of Interest

RV, APr, and AK were employed by the company Karkinos Healthcare Private Limited. The remaining authors declare that the research was conducted in the absence of any commercial or financial relationships that could be construed as a potential conflict of interest.

## Publisher's Note

All claims expressed in this article are solely those of the authors and do not necessarily represent those of their affiliated organizations, or those of the publisher, the editors and the reviewers. Any product that may be evaluated in this article, or claim that may be made by its manufacturer, is not guaranteed or endorsed by the publisher.
